# In-office hysteroscopic removal of retained or fragmented intrauterine device without anesthesia: a cross-sectional analysis of an international survey

**DOI:** 10.1007/s13304-022-01246-0

**Published:** 2022-02-05

**Authors:** Salvatore Giovanni Vitale, Attilio Di Spiezio Sardo, Gaetano Riemma, Pasquale De Franciscis, Luis Alonso Pacheco, Jose Carugno

**Affiliations:** 1 Obstetrics and Gynecology Unit, Department of General Surgery and Medical Surgical Specialties, University of Catania, Catania, Italy; 2 Department of Public Health, University of Naples “Federico II”, Naples, Italy; 3 Department of Woman, Child and General and Specialized Surgery, Obstetrics and Gynecology Unit, University of Campania “Luigi Vanvitelli”, Largo Madonna delle Grazie 1, 80138 Naples, Italy; 4 Unidad de Endoscopia Ginecológica, Centro Gutenberg, Hospital Xanit Internacional, Málaga, Spain; 5 Obstetrics and Gynecology Department, Minimally Invasive Gynecology Division, Miller School of Medicine, University of Miami, Miami, FL USA

**Keywords:** Intrauterine device, Hysteroscopy, Hysteroscopic removal, Copper IUD, LNG-IUS

## Abstract

To investigate about the opinions of gynecologists regarding the in-office hysteroscopic removal of retained or fragmented intrauterine device (IUD) without anesthesia. An online survey was made available to gynecologists who routinely performed in-office hysteroscopy. Five areas of interest were analyzed: average number of hysteroscopic procedures performed without anesthesia, availability on their local market of the different types of hormonal and non-hormonal IUDs, reasons for the hysteroscopic removal of the IUD, types of IUDs that were more commonly found retained or fragmented and, overall difficulty of the hysteroscopic removal. A total of 419 surgeons voluntarily responded the survey, of which 19 were excluded for not performing in-office hysteroscopy. The most commonly available IUD was the Levonorgestrel-based Mirena (Bayer Healthcare, Germany) or similar, (399/400, 99.7%), followed by Copper T (Paragard, CooperSurgical INC, United States) (397/400, 99.2%), Multiload (234/400, 58.5%) and Jaydess (Bayer Healthcare, Germany) (227/400, 56.7%). The intracavitary retention of the IUD with (44.5%, 178/400) and without (42.2%, 169/400) visible strings accounted as the most common reason for undergoing hysteroscopic IUD removal. Copper T IUD was the most common intracavitary retained (297/400, 74.2%) as well as fragmented device (236/400, 59.9%). The in-office hysteroscopic removal of the IUD was considered an easy procedure by almost all the operators (386/400, 96.5%). In-office hysteroscopy without anesthesia is seen as a feasible and easy approach to remove retained or fragmented IUDs inside the uterine cavity or cervical canal. While the Levonorgestrel-based IUD is the most commercialized, Copper T IUDs are the most commonly found retained or fragmented.

## Introduction

The use of long acting reversible contraception (LARC), especially the intrauterine devices (IUDs) has dramatically increased over the last thirty years. Globally, IUDs are one of the preferred form of contraception, with almost 20% of women worldwide using an IUD as their contraception of choice [1].

Even if contraception remains their main purpose, IUDs are also used for several non-contraceptive issues [2], especially with the advent of the levonorgestrel-releasing intrauterine system (LNG-IUS) device, which is considered a great treatment option for women with abnormal uterine bleeding and heavy menstrual bleeding, as well as dysmenorrhea [2, 3]. Similarly, LNG-IUS are used for the conservative treatment of atypical endometrial hyperplasia and early endometrial cancer following hysteroscopic resection in reproductive age women [4] as well as the treatment of adenomyosis and endometriosis [2]. LNG-based IUDs are commonly used by women desiring to use local hormonal contraception [5]. A non-hormonal intrauterine contraceptive option is the Copper IUD which is one of the most common LARC option used by young women [6]. There are many types of non-hormonal IUDs currently available in Europe, including the Copper T IUD, and the Multiload Copper IUD with a horse-shoe shape [7]. Also, the placement of a non-hormonal IUD after hysteroscopic lysis of adhesions or any other intrauterine adhesion generating procedure such as extensive myomectomy, is an effective strategy to avoid intrauterine adhesion formation and restore a normal anatomy of the uterine cavity [8, 9].

To date, starting from the introduction of the LNG-IUS in the 1990s, several studies reported that the most commonly used IUDs in America and Europe is the LNG-based IUD [10]. Nonetheless, IUDs are not risk-free, and several complications have been reported, including unintended expulsion, misplacement, and uterine perforation [11]. The vast majority of the misplaced IUDs are found inside the uterine cavity or cervix, while rarely, the IUD perforates the uterine serosa, migrating inside the peritoneal cavity [12]. Uterine perforation with an IUD is uncommon, but it may have serious consequences, including intra-abdominal bleeding, bowel or bladder perforation, and fistula formation, especially when IUDs migrate into the pelvic peritoneal space invading the adjacent organs [12]. Rarely, at the time of IUD removal, the device could break and fragments of the IUD could be left inside the uterine cavity.

For the removal of retained IUDs (with or without visible strings) or for the extraction of fragmented IUDs that are located inside the uterine cavity or the cervical canal, in-office hysteroscopy with or without anesthesia has been reported as a feasible way to retrieve them or, alternatively, to reposition them in the desired location [13–17].

The purpose of this cross-sectional study was to give insights on the in-office hysteroscopic removal of IUDs, capturing issues shared by gynecologists around the world and to determine the types of IUDs that more likely require the hysteroscopic approach for their removal.

## Methods

This Internet-based cross-sectional survey was conducted among gynecologists who report that they routinely performed in-office hysteroscopy without anesthesia as part of their gynecologic practice.

It was designed following the Helsinki Declaration and conformed to the Committee on Publication Ethics (COPE) guidelines. The protocol design, collection, analysis, and interpretation of data, as well as drafting, and subsequent revisions followed the Strengthening the Reporting of Observational Studies in Epidemiology (STROBE) Statement: guidelines for reporting observational studies [18].

Approval by an independent Institutional Review Board (IRB) was deemed not needed, since there were no women included and the study did not require interventions on the analyzed cohort.

The survey was conceptualized by the “Global Community of Hysteroscopy (GCH)”. It was constructed by the GCH scientific committee (A.D.S.S., L.A.S., J. C. and S.G.V.) and was reviewed by an expert panel of international researchers for content validity and reliability.

The survey was sent throughout email to all members of the GCH and the subscribers of the GCH mailing list. It was administered using the web-based program SurveyMonkey (SurveyMonkey, 2020). Participation was voluntary, with no monetary compensation, filling the survey was considered as consent to participate, and no participant identity could be identified.

To maintain anonymity, the only demographics-related question captured was the country in which the participant had their gynecologic practice. Answers were considered to refer to a respondent’s current practices. The survey was intended for gynecologists who personally performed hysteroscopic IUD removal.

The online survey comprised of 11 multiple choice questions. Questions centered on the following 5 content areas:

- The average number of in-office hysteroscopic procedures performed without anesthesia by the respondent.
- The availability on the local individual practice market of the different types of hormonal and non-hormonal IUDs
- The reasons why the removal of the IUD was performed hysteroscopically.
- The types of IUDs that were most commonly found retained with or without visible strings
- The overall difficulty of the in-office hysteroscopic IUD removal.

A 14-day response period was allowed before determining that the invited gynecologist was not willing to participate. The survey was emailed to surgeons up to three times before they were counted as non-respondents. Responses could not be traced to individual using internet data.

Statistical analysis was conducted using Stata 14.1 (Stata corp., College Station, TX, USA) and GraphPad Prism 8 (Graphpad, La Jolla, CA, USA). Descriptive data were computed for the primary analysis. For symmetrically distributed continuous variables, means, standard deviations (SDs) were reported, and the mean differences were analyzed using the t-test. Dichotomous data were depicted as absolute number and percentages. Differences in the proportions between the groups were evaluated by means of the Fisher’s exact or Chi square test for multiple comparisons, where appropriate. Statistical significance was defined at *p*-value (*p*) ≤ 05.

## Results

Four hundred nineteen gynecologists received and successfully fulfilled the survey. Respondents reported that their gynecologic practice was located mainly in South America (35.6%; 149/419) and Europe (32.0%; 134/419), followed by North and Central America (14.3%; 60/419), Asia (12.6%; 53/419), and Africa (5.5%; 23/419). Nineteen surgeons were excluded from the analysis since they were not the operators of the IUD removal.

All the participants (100%, 400/400) performed the hysteroscopic removal in the in-office setting. Similarly, all the operators performed the procedures without the use of anesthesia, using a vaginoscopic approach without using a speculum or tenaculum to visualize and hold the uterine cervix.

Regarding the number of hysteroscopic procedures performed by the 400 surgeons included in the final analysis, 52 (13.0%) reported that they work in a center performing more than 1000 hysteroscopies each year, the 22.5% (90 operators) work in centers performing between 500 and 1000 procedures, while the 37.8% (151 out of 400) between 100 and 500, and 107 (26.7%) work in centers performing on average less than 100 hysteroscopic procedures each year.

**Fig. 1:**
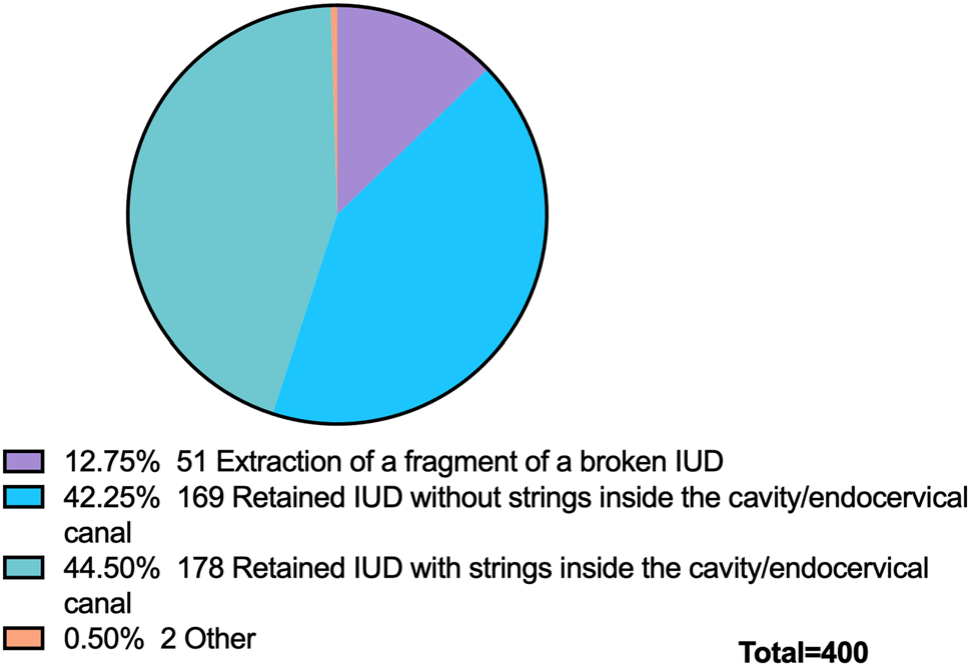
Most common reason for in-office hysteroscopic removal of IUD

Several reasons led the operators to perform the hysteroscopic removal of the IUD, including the intrauterine retention without or with visible strings, as well its fragmentation. The distribution of the most common reasons (Intracavitary retained IUD with (44.5%, 178/400) and without (42.2%, 169/400) visible strings) for using hysteroscopy for the removal is depicted in Fig. 1.

**Fig. 2:**
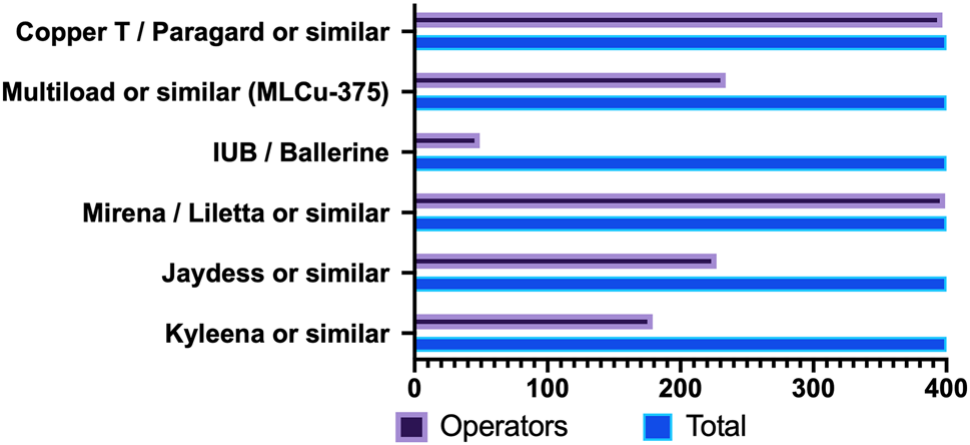
Commercially available IUD according to the provider’s practice location

The availability of IUDs varied across the operators’ practice geographic location. The most commonly available IUD was the LNG-IUS Mirena (Bayer Healthcare, Germany) or similar, which was available for 399 out of 400 participants (99.7%), followed by Copper T (Paragard, CooperSurgical INC, United States) or similar (397/400, 99.2%), Multiload (234/400, 58.5%), Jaydess (Bayer Healthcare, Germany) (227/400, 56.7%), Kyleena (Bayer Healthcare, Germany) (179/400, 44.7%), and IUB-Ballerine (OCON Medical LTD, Israel) (49/400, 12.3%) (Fig. 2).

**Fig. 3:**
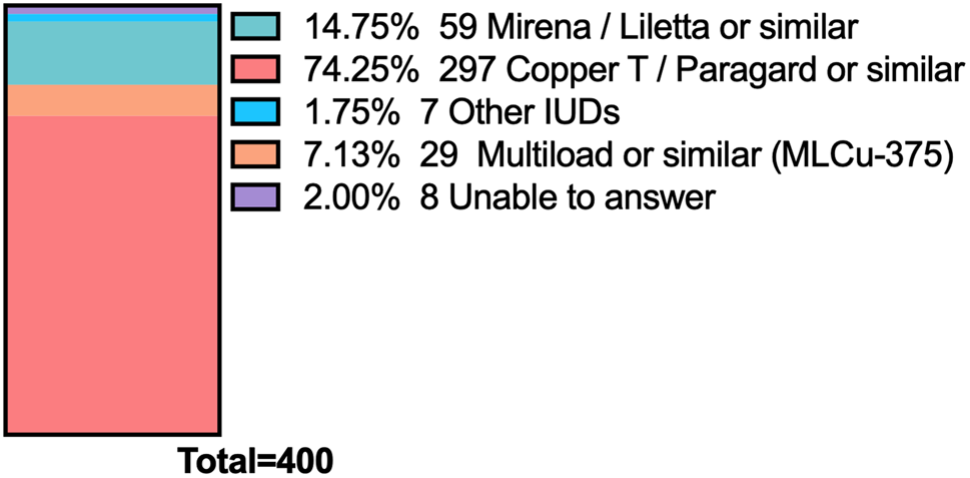
Most common IUDs found retained without visible strings inside the uterus

**Fig. 4:**
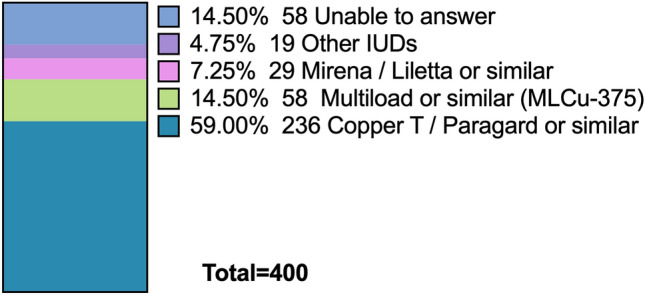
Most common IUDs found fragmented inside the uterus

Regarding the type of IUD that was most commonly found retained inside the uterine cavity without visible strings was the Copper T IUD (297/400, 74.2%), followed by Mirena^®^ (59/400, 14.8%) and Multiload^®^ (29/400, 7.3%) (Fig. 3). Meanwhile, in case of fragmented IUD, participants also reported that Copper T IUDs was the most common type (236/400, 59.0%), followed by the Multiload or similar (58/400, 14.5%) (Fig. 4).

To investigate the differences of opinions between participants, when divided according to the number of hysteroscopic procedures that they indicated on average performing per year, we conducted a subgroup analysis for the most frequent indication for the in-office hysteroscopic removal, the most frequently extracted IUD in case of intracavitary retention without visible strings and the most common IUD fragmented inside the uterine cavity. Table 1 shows that there were statistically significant differences regarding the most frequent indication for hysteroscopy. A retained IUD with strings inside the cavity or the endocervical canal accounted for the highest number of hysteroscopies performed in centers performing more than 1000 or between 500 and 1000 procedures each year (47/90 and 33/52 vs 34/107 and 62/151 respectively, *p* < 0.001). Conversely, IUD without visible strings inside the cavity were most frequently extracted in centers performing between 500 and 100 or less than 100 in-office hysteroscopies in one year (63/107 and 67/151 vs 24/90 and 12/52 respectively, *p* < 0.001) (Table 1).

**Table 1:** Subgroup analysis according to the average number of hysteroscopic procedures performed in one year by each provider

Hysteroscopic procedures performed in one year (n, %)		Less than 100 (107, 26.7)	Between 100 and 500 (151, 37.7)	Between 500 and 1000 (90, 22.5)	More than 1000 (52, 13)	*p* value
*Most frequent indication for hysteroscopy*						< 0.001
Retained IUD with visible strings inside the cavity/endocervical canal (%)		34 (31.8)	62 (41.1)	47 (77.8)*	33 (63.5)*	
Retained IUD without visible strings (%)		63 (58.9)°	67 (44.4)°	24 (11.0)	12 (23.1)	
Extraction of a fragment of IUD (%)		9 (8.4)	18 (11.9)	17 (5.6)	6 (11.5)	
Other (%)		1 (1.0)	4 (2.6)	2 (5.6)	1 (1.9)	
*In a case of a retained IUD without visible strings, which type of IUD is the most frequently extracted?*						0.496
Copper T (%)		85 (79.4)	109 (71.1)	63 (70.0)	37 (71.1)	
Mirena (%)		13 (12.1)	23 (15.2)	14 (15.6)	8 (15.4)	
Multiload (%)		6 (5.6)	13 (8.6)	7 (7.8)	3 (5.8)	
Other (%)		3 (2.9)	6 (5.1)	6 (6.6)	4 (7.7)	
*In a case of fragmented IUD, which type is the one most frequently extracted?*						0.174
Copper T (%)		67 (55.8)	88 (58.2)	64 (71.1)	33 (63.4)	
Mirena (%)		8 (6.7)	15 (9.9)	3 (3.3)	3 (5.8)	
Multiload (%)		2 (1.7)	31 (20.5)	15 (16.7)	10 (19.2)	
Other (%)		9 (7.5)	1 (0.7)	2 (2.2)	6 (11.6)	
Unable to answer (%)		34 (28.3)	15 (10.7)	6 (6.7)	0	

*p < 0.001 vs “Less than 100” and “Between 100 and 500”

°*p* < 0.001 vs Between “500 and 1000” and “More than 1000”

When asked to categorize the difficulty of the procedure, most of the participants described the in-office hysteroscopic removal of the IUD as an easy procedure (386/400, 96.5%).

## Discussion

This cross-sectional analysis of an international survey, aiming to understand the current opinion of gynecologists about the hysteroscopic removal of IUDs, showed that, although the LNG-IUS was the most used IUD device worldwide and, that the Copper T was the IUD that was most found retained or fragmented inside the uterine cavity.

Why is it important to remove a fragmented or misplaced IUD? The presence of a misplaced Copper IUD or LNG-IUS increases the risk of contraceptive failure [1]. A recent study found a significant difference in pregnancy rates between women using a Copper versus LNG-IUS devices, with a significant lower pregnancy rate in those using the LNG-IUS [19]. This could be due to the different mechanisms of action of these two types of IUDs. The contraceptive effect of copper IUD is based on the instauration of a local endometrial inflammation as well as on the blocking effect of copper (Cu) ions on spermatozoa motility [10]. On the contrary, the mechanism of action of the LNG-IUS is based on causing endometrial atrophy and thickening of the cervical mucus [20]. An LNG-IUS does not have significant systemic effects since the plasma levels of LNG are low and its impact on the ovarian function is minimal. It is known that a misplaced LNG-IUS, provides similar circulating levels of LNG than those retrievable in patients receiving combined oral contraceptives [21]. It has been hypothesized that these levels are enough to prevent ovulation and, therefore, conception. [21] Moreover, in-vivo studies have shown that, due to the anti-inflammatory properties of LNG, when LNG-IUDs are displaced into the peritoneal cavity, the peritoneal adhesion formation is minimal [10].

Retained IUDs or fragments of it when broken at the time of removal can be retrieved in the office using several approaches (i.e. a cervical brush attempting to retrieve the strings, thread retrievers, IUD hooks), with or without ultrasound guidance. Generally, these procedures cause discomfort to the patient, are difficult for the operator and often lead to failure to remove the IUD [22]. In such cases, women could undergo in-office hysteroscopy to retrieve the misplaced IUD. The hysteroscopic removal of misplaced IUD, or fragments of it, was categorized as easy by the majority of gynecologists participating in this survey. Moreover, the minimal patient’s discomfort and the high success rate of retrieval with in-office hysteroscopy without anesthesia reveals that is a feasible and effective approach for the retrieval of an IUD with not visible strings [19, 23, 24].

It is important to highlight that considering the higher number of complications in patients using T-Shaped Copper IUDs, compared to the LNG-IUS or non-T shaped Copper IUDs, could justify favoring the use of LNG-IUS or the Copper Multiload IUDs, with horse-shoe design and flexible arms, which were reported to have significantly lower rates of perforation and expulsion in two previous studies [25, 26].

We acknowledge that this cross-sectional analysis has several limitations. First, although the total number of participants was enough to draw a conclusion, there were several geographic areas (i.e., Africa), in which respondents were too few to obtain a meaningful response. Moreover, the majority of answers were related to Copper T IUDs or Mirena IUD; this should be due to the fact that other LNG-IUS and IUDs are not globally available, as revealed in this survey. Nonetheless, this survey depicts self-reported data from operators and their replies may be interpreted as estimates rather than objectively confirmed data from clinical practice, for which a prospectively collection of data is mandatory. These survey estimations are also subjected to nonresponse bias, since surgeons who kindly replied to the invitation could be intrinsically different from operators who declined the invitation.

Despite the abovementioned limitations, this cross-sectional analysis is a first look about the feedbacks of an international gynecologic community concerning their opinions on the hysteroscopic management of misplaced or fragmented IUDs.

## Conclusions

In-office hysteroscopic removal of a retained or fragmented IUD is a feasible and effective procedure reported as easy by gynecologists from all over the world. A retained IUD device without visualizable strings is the most common indication for hysteroscopic removal. Copper T IUDs are reported to be the most commonly found retained or fragmented inside the uterine cavity.

## Data Availability

The data that support the results of this cross-sectional analysis are available from the corresponding author, upon reasonable request.
